# Machine Learning Models Cannot Replace Screening Colonoscopy for the Prediction of Advanced Colorectal Adenoma

**DOI:** 10.3390/jpm11100981

**Published:** 2021-09-29

**Authors:** Georg Semmler, Sarah Wernly, Bernhard Wernly, Behrooz Mamandipoor, Sebastian Bachmayer, Lorenz Semmler, Elmar Aigner, Christian Datz, Venet Osmani

**Affiliations:** 1 Department of Internal Medicine, General Hospital Oberndorf, Teaching Hospital of the Paracelsus Medical University Salzburg, 5020 Salzburg, Austria; georg.semmler@hotmail.com (G.S.); sarah@wernly.net (S.W.); bachmayer@live.at (S.B.); lorenz.semmler@meduniwien.ac.at (L.S.); 2 Division of Gastroenterology and Hepatology, Department of Internal Medicine III, Medical University of Vienna, 1090 Vienna, Austria; 3 Second Department of Medicine, Paracelsus Medical University Salzburg, 5020 Salzburg, Austria; bernhard@wernly.net; 4 Fondazione Bruno Kessler Research Institute, 38123 Trento, Italy; bmamandipoor@fbk.eu; 5 First Department of Medicine, Paracelsus Medical University Salzburg, 5020 Salzburg, Austria; e.aigner@salk.at

**Keywords:** machine learning, artificial intelligence, colorectal adenoma, colorectal cancer, advanced adenoma, screening

## Abstract

Screening for colorectal cancer (CRC) continues to rely on colonoscopy and/or fecal occult blood testing since other (non-invasive) risk-stratification systems have not yet been implemented into European guidelines. In this study, we evaluate the potential of machine learning (ML) methods to predict advanced adenomas (AAs) in 5862 individuals participating in a screening program for colorectal cancer. Adenomas were diagnosed histologically with an AA being ≥ 1 cm in size or with high-grade dysplasia/villous features being present. Logistic regression (LR) and extreme gradient boosting (XGBoost) algorithms were evaluated for AA prediction. The mean age was 58.7 ± 9.7 years with 2811 males (48.0%), 1404 (24.0%) of whom suffered from obesity (BMI ≥ 30 kg/m²), 871 (14.9%) from diabetes, and 2095 (39.1%) from metabolic syndrome. An adenoma was detected in 1884 (32.1%), as well as AAs in 437 (7.5%). Modelling 36 laboratory parameters, eight clinical parameters, and data on eight food types/dietary patterns, moderate accuracy in predicting AAs with XGBoost and LR (AUC-ROC of 0.65–0.68) could be achieved. Limiting variables to established risk factors for AAs did not significantly improve performance. Moreover, subgroup analyses in subjects without genetic predispositions, in individuals aged 45–80 years, or in gender-specific analyses showed similar results. In conclusion, ML based on point-prevalence laboratory and clinical information does not accurately predict AAs.

## 1. Introduction

Colorectal cancer (CRC) is the third most frequent malignancy and the fourth most common cause of death due to cancer worldwide [1]. Several screening modalities for CRC have been proposed including colonoscopy, flexible sigmoidoscopy, or (guaiac/immunochemical) fecal occult blood testing (FOBT) [2]. Specifically, European guidelines highlight the evidence that screening based on FOBT reduces mortality from CRC while less evidence exists for the efficacy, positive risk/benefit ratio, and cost-effectiveness of opportunistic screening colonoscopy [3]. However, detection rates of polyps and adenomas are regarded higher in screening programs using colonoscopy [4], indicating that this might be the most sensitive option for an individual patient.

Several risk-prediction tools have been developed to pre-classify patients as those with an advanced adenoma (AA) or CRC and those without [5,6,7,8]. However, when assessing the “area under the receiver operating characteristic curve” (AUC-ROC) for correct classification, these approaches only yielded a moderately accurate prediction (AUC-ROC for AA: 0.61–0.71) [5,6]. To optimize risk prediction in medical science, machine learning (ML) is a novel and increasingly popular tool [9,10]. Despite potentially superior performance compared to regular statistical methods, ML has not been applied for CRC screening. Thus, the aim of this study was to evaluate the potential of ML for the prediction of an AA, a precursor lesion of CRC, in an Austrian screening colonoscopy cohort.

## 2. Materials and Methods

### 2.1. Population and Study Design

Overall, 6129 consecutive individuals participating in an Austrian screening program for colorectal cancer (i.e., cross-sectional cohort study) between 2010 and 2020 were eligible for inclusion in this analysis. This included 267 patients with a history of CRC, symptomatic patients, and those with insufficient colonoscopies (i.e., not reaching the ascending colon) were excluded, resulting in a study cohort of 5862 individuals. 

Subjects were screened for CRC by colonoscopy and further characterized using laboratory and clinical data that were obtained the day before. They also completed a detailed questionnaire on lifestyle and dietary habits. The study design and details of the clinical and biochemical work-up included subjects that have been reported previously [11] and are included in the supplement. 

### 2.2. Colonoscopy

Screening for colorectal cancer was performed by colonoscopy according to the published guidelines [3]. All polyps were sent for histologic analysis and were characterized based on their macroscopic and histologic results. Polyps were classified as hyperplastic polyps, adenomas, advanced adenomas, and CRC. An adenoma was defined as being advanced if (1) its size was ≥ 1cm, (2) high-grade dysplasia was present, or (3) villous features were seen histologically [12]. Additional definitions are provided in the Supplementary Materials.

### 2.3. Machine Learning Model Development and Evaluation

We divided the data randomly into two groups: 80% for the model development and internal validation cohort and 20% for the test cohort. The test cohort was kept apart and was not used in any way during model development. The development dataset was further divided into the model derivation dataset (80%), while the remaining data (20%) were used to fine-tune the hyperparameters of the models.

Internal validation on the development dataset was carried out using 5-fold, stratified cross-validation with 10 times repetition. The best performing model in terms of the AUC-ROC from the internal validation cohort was then evaluated on the test cohort.

Based on this methodology, we evaluated the performance of two algorithms, namely logistic regression (LR) and extreme gradient boosting (XGBoost) [13]. The missing values were imputed with median and mode for numerical and categorical variables, respectively. Additionally, we normalized numerical values between 0 and 1.

### 2.4. Statistical Analyses

We assessed the performance of each model by computing the AUC-ROCs. For each model, we provide performance metrics, including sensitivity, specificity, positive predictive value (PPV), and negative predictive value (NPV), including 95% confidence intervals to quantify uncertainty. A two-sided *p*-value < 0.05 was considered statistically significant.

## 3. Results

### 3.1. Patient Characteristics

Overall, the mean age of participants was 58.7 *±* 9.7 years, with 2811 males (48.0%) and 3051 females (52.0%; Table 1). A total of 659 individuals (11.2%) had a first-degree relative with a history of CRC, while 1404 (24.0%) suffered from obesity (BMI *≥* 30 kg/m²), 871 (14.9%) from diabetes, and 2095 (39.1%) from metabolic syndrome. We detected a hyperplastic polyp in 1737 (29.6%), an adenoma in 1884 (32.1%), an AA in 437 (7.5%), and CRC in 45 subjects (0.8%).

### 3.2. Prediction of Advanced Adenoma Based on Machine Learning Models

Initially, we investigated whether machine learning models (LR and XGBoost) were able to predict AAs from the clinical and laboratory variables of the respective patient. We, therefore, included 36 laboratory parameters, eight clinical parameters (including age, sex, data on smoking behaviour, hepatic steatosis, family history of CRC, and data on medication, e.g., acetylsalicylic acid, statins, and ACE-inhibitors), and data on eight food types/dietary patterns including the consumption of alcohol, coffee, red meat, sugar-sweetened beverages (SSB), fruits, vegetables, and fast-food. 

**Figure 1:**
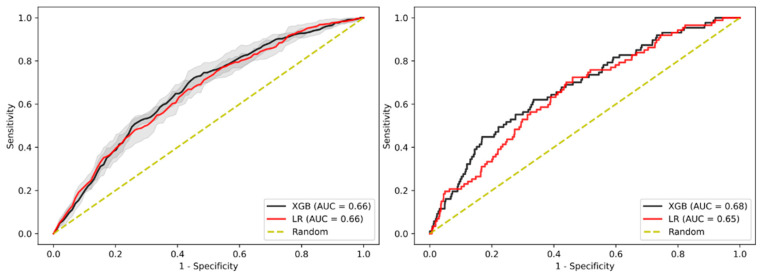
AUC-ROC performance of the XGBoost-based model and Logistic Regression (LR)-based model to identify patients with advanced adenoma (AA) for internal validation cohort (**left**) and test cohort (**right**). Confidence intervals of 95% are shown in shaded grey.

In terms of predictive performance, both models showed a moderate accuracy for identifying patients with AAs in the test cohort, with XGBoost showing AUC-ROC of 0.68 and LR an AUC-ROC of 0.65. The internal validation cohort showed slightly lower performance with an AUC-ROC of 0.66 (95% CI: 0.650–0.663) and 0.66 (95% CI: 0.658–0.668), respectively, as shown in Figure 1 and Supplementary Table S1. There was no statistically significant difference between the performance of LR and XGBoost in the internal validation cohort.

### 3.3. Sensitivity Analysis Using Only Established Risk Modifiers

**Figure 2:**
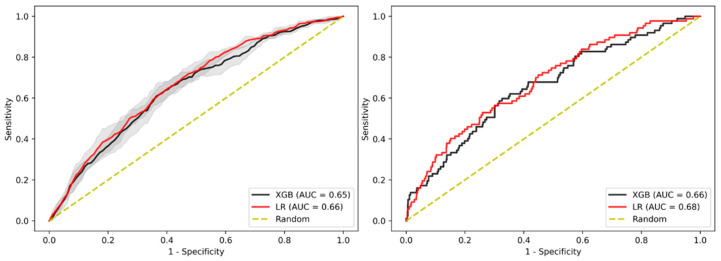
AUC-ROC performance of the XGBoost-based model and LR-based model to identify patients with AA when selecting only variables with a suspected pathophysiological influence of AA risk for the internal validation cohort. Internal validation cohort (**left**) and test cohort (**right**). Confidence intervals of 95% are shown in shaded grey.

Next, we conducted a sensitivity analysis to investigate whether a subset of variables previously associated with AAs provides an increase in predictive performance. This subset of variables included both clinical variables (systolic blood pressure, serum glucose, C-reactive protein, and low-density lipoprotein cholesterol) as well as lifestyle variables (family history of CRC, smoking status, and alcohol use), in addition to demographic variables (age, sex, and BMI). In the test cohort, LR outperformed XGBoost with an AUC-ROC of 0.68 and 0.66, respectively, while there was no difference in performance between these models in the internal validation cohort when using LR (AUC-ROC of 0.66 (95% CI: 0.660–0.670)) or XGBoost (AUC-ROC of 0.65 (95% CI: 0.642–0.658)), as shown in Figure 2 and Supplementary Table S2.

### 3.4. Sensitivity Analysis on Patients without Family History

**Figure 3:**
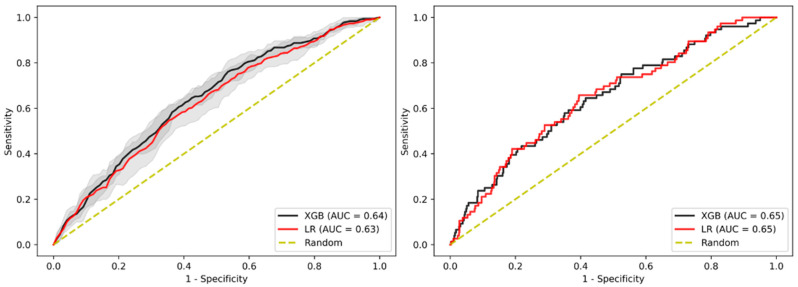
AUC-ROC performance of the XGBoost-based model and LR-based model to identify patients with AA when removing patients with a known family history risk. Internal validation cohort (**left**) and test cohort (**right**). Confidence intervals of 95% are shown in shaded grey.

This analysis focused on predicting AAs only on subjects without a family history of AAs in first-degree relatives while using all the available variables. This approach did not reveal significant differences between this sub-cohort and the overall cohort in the internal validation cohort with an AUC-ROC of 0.64 for XGBoost and 0.63 for LR, while the test cohort showed decreased performance for XGBoost, having an AUC-ROC of 0.65 for both models (Figure 3, Supplementary Table S3).

### 3.5. Sensitivity Analysis on Age

We also investigated whether the prediction of AAs in a specific age range would improve prediction performance. For this analysis, we defined a sub-cohort of patients between the ages of 45 and 80 at the time of examination since these subjects are the primary target population for CRC screening.

**Figure 4:**
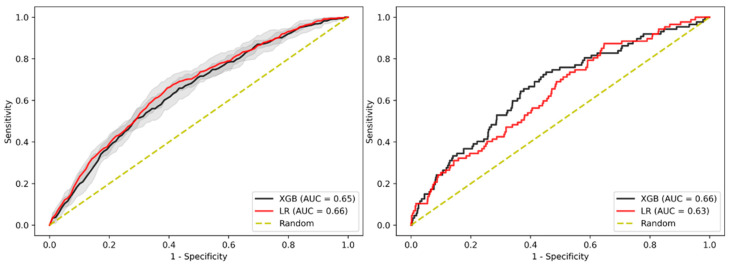
AUC-ROC performance of the XGBoost-based model and LR-based model to identify patients with AA when restricting cohort age range between (and including) 45 and 80 years. Internal validation cohort (**left**) and test cohort (**right**). Confidence intervals of 95% are shown in shaded grey.

The results in Figure 4 showed similar performance to the overall cohort with an AUC-ROC of 0.65 (95% CI 0.640–0.652) for XGBoost and 0.66 (95% CI (0.656–0.668) for LR in the internal validation and an AUC-ROC of 0.66 for XGBoost and 0.63 for LR when evaluated on the test cohort (Supplementary Table S4).

### 3.6. Sensitivity Analysis on Gender

**Figure 5:**
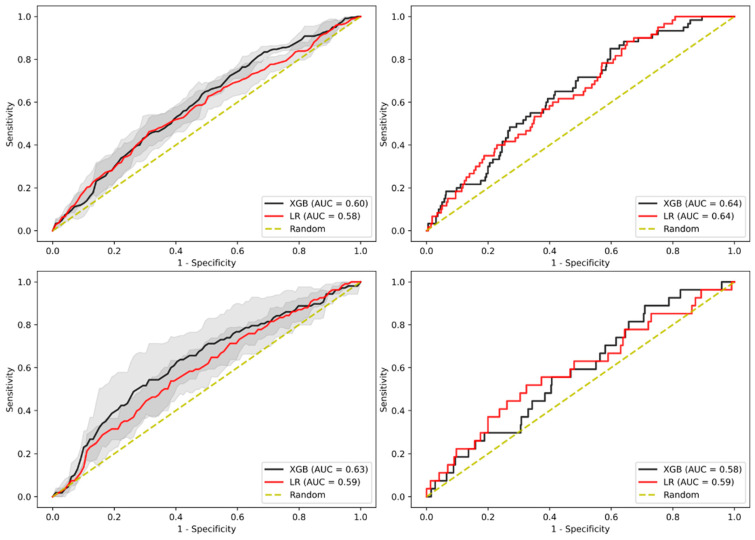
AUC-ROC performance of the XGBoost (XGB)-based model and LR-based model to identify patients with AA when separating cohort between male (**top**) and female patients (**bottom**). Internal validation cohort (**left**) and test cohort (**right**). Confidence intervals of 95% are shown in shaded grey.

Previous work has shown that males are more likely to develop AAs and CRC than females [14,15]. As such, we divided the overall cohort across gender and derived as well as evaluated two separate models for each gender. As can be seen in Figure 5, there were no significant differences between genders in terms of AUC-ROC performance, where the model-derived and internally validated in the male cohort achieved an AUC-ROC of 0.60 (95% CI 0.587–0.604) for XGBoost and 0.58 (95% CI 0.566–0.589) for LR as well as an AUC-ROC of 0.64 for both models in the test cohort. On the other hand, the female cohort-derived model achieved an AUC-ROC of 0.63 (95% CI 0.603–0.658) for XGBoost and 0.59 (95% CI 0.579–0.604) for LR in internal validation as well as an AUC-ROC of 0.58 for XGBoost and 0.59 for LR in the test cohort (Supplementary Table S5).

### 3.7. Sensitivity Analysis on Sub-Cohort with Advanced Adenomas Only

**Figure 6:**
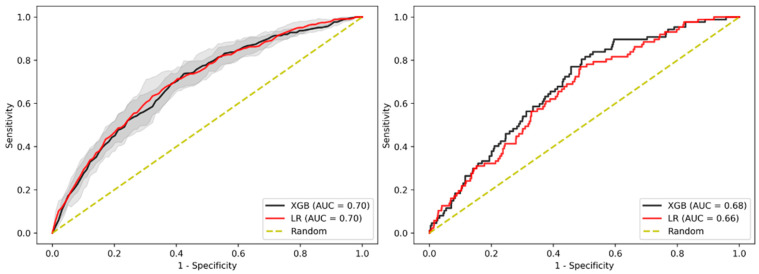
AUC-ROC performance of the XGBoost-based model and LR-based model to identify patients with AA when removing patients with adenomas. Internal validation cohort (**left**) and test cohort (**right**). Confidence intervals of 95% are shown in shaded grey.

Since the adenoma-carcinoma sequence is considered a somewhat continuous process, we performed an analysis removing individuals with adenomas but without AAs to test whether our models could better discriminate between the target population (AA) and completely “healthy” individuals in terms of CRC. Derivation and evaluation of the models on this sub-cohort showed an AUC-ROC of 0.70 (95% CI 0.685–0.709 and 0.693–0.711) for XGBoost and LR models in the internal validation, and an AUC-ROC of 0.68 for XGBoost and 0.66 for LR in the test cohort (Figure 6, Supplementary Table S6).

### 3.8. Imputation Method

Finally, we performed the multivariate imputation method based on a gradient boosting regressor and classifier to impute numerical and categorical data values that were missing in the dataset, along with applying log transformation to reduce the skewness of the original data and normalizing the numerical variables using z-score and linear scaling. However, these approaches did not yield a higher accuracy for the classification of AAs (data not shown). 

## 4. Discussion

In this study, we investigated whether ML algorithms could improve the accuracy of AA prediction. We found that two established ML methods (LR and XGBoost) only provide moderate accuracy to correctly diagnose an AA based on 52 readily available laboratory parameters, clinical parameters, and dietary patterns.

Apart from evidence that is currently emerging on the cost-effectiveness of screening colonoscopies including a reduction in CRC mortality [16,17,18], it is also regarded as the gold-standard for CRC screening due to its high sensitivity and the advantages it provides to directly remove or histologically analyse suspicious lesions. However, participation rates vary, and mild side-effects (i.e., abdominal pain), as well as the fear of adverse events, may prevent individuals from participating in screening colonoscopies [19]. Especially in asymptomatic individuals, these barriers significantly hamper CRC screening programs [20]. Moreover, an ongoing controversy exists on the usefulness of screening colonoscopies in subjects less than 50 years of age [15,21]. While still being cost-effective, a greater benefit could be assumed by increasing participation rates for unscreened older and higher-risk persons [22]. For all these issues, tests that increase the pre-test probability of a positive finding on a screening colonoscopy may definitely be useful. By identifying those with a higher likelihood of AAs, these individuals could be invited earlier and more frequently while simultaneously increasing their willingness to participate (demonstrated for FOBT [23]). Resources could be re-distributed from those least likely to those most likely to benefit from a colonoscopy [22]. Finally, certain aspects during the colonoscopy could be individualized. On the one hand, the time for colon exploration by the gastroenterologist could be extended in patients with a higher pre-test probability. On the other hand, gastroenterologists could also pay closer attention to a patient with a higher probability of having an advanced adenoma.

Unfortunately, none of the published scores were yet able to provide meaningful risk estimations for AAs or CRC, and none of the published scores have been included in guideline recommendations [5,6,7]. Our models based on two ML approaches yielded a moderate diagnostic accuracy to correctly classify individuals with or without an AA (AUC-ROC~0.66). Although this seems disappointing, previous models have yielded a similar accuracy in predicting CRC or AAs. Smith et al. [7] investigated 16 scores and found that they were able to discriminate between individuals who subsequently developed CRC and those who did not with moderate accuracy (most c-statistic estimates ranged between 0.65 and 0.71). Similar results were reported from other models that only reached moderate accuracy (assessed as the AUC-ROC for prediction of five-year CRC risk of <0.70) [8]. For AA prediction, results were largely comparable with an AUC-ROC of risk scores ranging from 0.62 to 0.77 in individual studies, from 0.57 to 0.65 in validation cohorts, and from 0.61 to 0.71 in a meta-analysis [5,6]. In addition, a recently proposed polygenic risk score for prediction of CRC yielded an AUC-ROC of 0.65 [24], while another five-marker blood test (carcinoembryonic antigen + anti-p53 + osteopontin + seprase + ferritin) only had an AUC-ROC of 0.56 [25]. 

In view of the literature, our presumably imperfect results can be interpreted as follows. First, more accurate risk prediction might not be possible with the available (ordinary) laboratory and clinical data since they only cover a limited part of CRC risk. Interestingly, several novel aspects of CRC prediction have been recently proposed, including cell-free DNA [26] and the gut microbiome [27], which can potentially improve risk prediction when applied to CRC screening.

Second, the question arises whether achieving a high AUC-ROC or c-statistic is a valid approach to assess the utility of an additional risk prediction tool for CRC screening. Achieving higher accuracy for correct classification in a whole cohort may not necessarily be a patient-centred outcome for a screening test. In contrast, any simple test that increases the pre-test probability of AAs for CRC screening may still be a valuable tool since alternatives are scarce. In this regard, our algorithm still identifies individuals at higher risk for AAs, which could substantially improve both screening adherence from the patient perspective and also adherence to quality measures for colonoscopies from the endoscopist perspective. 

Finally, Aleksandrova et al. [28] recently proposed the “LiFeCRC” model that focuses on lifestyle factors for predicting future CRC. This approach yielded moderate accuracy with a c-statistic estimate of 0.70, indicating that lifestyle factors indeed play a certain role in CRC development. However, we deliberately chose to focus on AA prediction as to the outcome-of-interest for several reasons. From a clinical perspective, AAs convey a significant risk of CRC development and need more stringent follow-up [12,29]. While the prediction of CRC might also be possible, the number of cases is significantly lower in a screening population. Thus, accurate risk prediction for CRC with the premise of not having false negative cases would consecutively lead to a high number of false-positive cases. On the other hand, false negative results in terms of cancer prediction might also be dangerous in terms of giving the patients a false sense of security. Thus, AA prediction seems more reasonable to us.

Apart from gastroenterology, machine learning has been gaining increasing popularity in many different medical fields. For instance, Khera et al. [30] compared the predictive values of machine-learning-based models with LR for in-hospital death among patients who were hospitalized for acute myocardial infarction. Interestingly, their results were similar to our study. When using the same data input, ML algorithms did not substantially improve the prediction of death compared to simple LR. One explanation for this might be that these algorithms need a significant number of variables to outperform LR. Therefore, we also added as many variables as was sensible for our initial approach. Apart from the quantity of variables, the quality and granularity of variables might also improve the respective model. Thus, it would be conceivable that a more complex ML algorithm (such as XGBoost versus LR) would benefit from a more complex dataset. For example, longitudinal laboratory and clinical data from multiple examinations conducted over several years could improve the predictive power of ML since they comprehensively cover the lifestyle of an individual.

Our results could also be interpreted as suggesting that other, yet unidentified, factors significantly influence CRC risk, such as genetics. Importantly, our dataset included a relevant number of cardiometabolic risk factors and yet suggested a relatively low predictive power based on this information. Interestingly, our approach, when restricted to the variables that convey a certain risk for CRC, yielded similar results compared to the approach using all the variables. This confirms that these parameters indeed have certain risk-modulating properties, to which other easily available variables add only minimal information.

One strength of this study is the broad characterization of patients that allowed us to include lifestyle parameters that are rarely available. However, a limitation of this study is the cross-sectional design for which ML might be less suitable compared to longitudinal data assessed as multiple timepoints. Another clear limitation is the lack of an analysis of the prevalence of sessile serrated lesions. However, these lesions were only recently recognized as their own entity and were not systematically assessed over the course of this study. Thus, we abstained from specific analyses on these polyps. In addition, as with any other screening cohort (not only with colonoscopy and FOBT screening, but also general screening for any disease), our cohort may be biased towards healthy volunteers. Finally, data on previous screening colonoscopies without any abnormalities were not fully documented in selected patients.

## 5. Conclusions

In conclusion, we show that ML based on point-prevalence laboratory and clinical information does not significantly improve risk prediction for AAs compared to other conventional statistical methods, highlighting that these predictive models are insufficient to replace current CRC screening programs. However, given the burden that CRC contributes to cancer-related mortality, algorithms based on individualized risk estimation are needed to improve CRC screening efficiency and accuracy.

## Figures and Tables

**Table 1:** Patient characteristics.

Variables	Overall, *n* = 5862
Age, years	58.7 ± 9.7
Sex	
Male	2811 (48.0%)
Female	3051 (52.0%
Obesity	1404 (24.0%)
BMI, kg/m²	27.2 ± 4.7
Metabolic syndrome ^1^	2095 (39.1%)
Hypertension	3272 (55.8%)
Systolic BP, mmHg	133 ± 19
DM	871 (14.9%)
Fatty liver disease ^2^	2613 (44.8%)
Ever/Current smoker	2898 (49.4%)
First degree relative with history of CRC	659 (11.2%)
Any HP	1737 (29.6%)
Any adenoma	1884 (32.1%)
Any AA	437 (7.5%)
CRC	45 (0.8%)
Advanced lesion ^3^	462 (7.9%)

^1^ Available in 5364 patients; ^2^ Available in 5832 patients; ^3^ Advanced adenoma (AA) and colorectal cancer (CRC).

## Data Availability

The data are available from the authors upon request.

## Supplementary Materials

The following are available online at https://www.mdpi.com/article/10.3390/jpm11100981/s1, Table S1: Detailed characteristics of the logistic regression (LR) and XGBoost-based models to predict advanced adenoma (AA). Table S2: Detailed characteristics of the logistic regression (LR) and XGBoost-based models to predict advanced adenoma (AA) when only selecting variables with suspected pathophysiological influence on AA risk. Table S3: Detailed characteristics of the logistic regression (LR) and XGBoost-based models to predict advanced adenoma (AA) when only selecting patients without family history. Table S4: Detailed characteristics of the logistic regression (LR) and XGBoost-based models to predict advanced adenoma (AA) when only selecting patients in specific age range between 45 and 80 at the time of examination. Table S5: Detailed characteristics of the logistic regression (LR) and XGBoost-based models to predict advanced adenoma (AA) when separating male and female cohorts. Table S6: Detailed characteristics of the logistic regression (LR) and XGBoost-based models to predict advanced adenoma (AA) when excluding individuals with any adenoma others than AA.

## References

[1] Arnold M., Sierra M.S., Laversanne M., Soerjomataram I., Jemal A., Bray F. (2017). Global patterns and trends in colorectal cancer incidence and mortality. Gut.

[2] Bénard F., Barkun A.N., Martel M., Von Renteln D. (2018). Systematic review of colorectal cancer screening guidelines for average-risk adults: Summarizing the current global recommendations. World J. Gastroenterol..

[3] von Karsa L., Patnick J., Segnan N., Atkin W., Halloran S., Saito H., Sauvaget C., Scharpantgen A., Schmiegel W., Senore C. (2013). European guidelines for quality assurance in colorectal cancer screening and diagnosis: Overview and introduction to the full supplement publication. Endoscopy.

[4] Benson V.S., Atkin W.S., Green J., Nadel M.R., Patnick J., Smith R.A., Villain P., on behalf of the International Colorectal Cancer Screening Network (2012). Toward standardizing and reporting colorectal cancer screening indicators on an international level: The international colorectal cancer screening network. Int. J. Cancer.

[5] Peng L., Weigl K., Boakye D., Brenner H. (2018). Risk Scores for Predicting Advanced Colorectal Neoplasia in the Average-risk Population: A Systematic Review and Meta-analysis. Am. J. Gastroenterol..

[6] Peng L., Balavarca Y., Weigl K., Hoffmeister M., Brenner H. (2019). Head-to-Head Comparison of the Performance of 17 Risk Models for Predicting Presence of Advanced Neoplasms in Colorectal Cancer Screening. Am. J. Gastroenterol..

[7] Smith T., Muller D., Moons K.G.M., Cross A.J., Johansson M., Ferrari P., Fagherazzi G., Peeters P.H.M., Severi G., Hüsing A. (2018). Comparison of prognostic models to predict the occurrence of colorectal cancer in asymptomatic individuals: A systematic literature review and external validation in the EPIC and UK Biobank prospective cohort studies. Gut.

[8] Usher-Smith J., Harshfield A., Saunders C., Sharp S.J., Emery J., Walter F., Muir K., Griffin S.J. (2018). External validation of risk prediction models for incident colorectal cancer using UK Biobank. Br. J. Cancer.

[9] Wernly B., Mamandipoor B., Baldia P., Jung C., Osmani V. (2021). Machine learning predicts mortality in septic patients using only routinely available ABG variables: A multi-centre evaluation. Int. J. Med. Inform..

[10] Goecks J., Jalili V., Heiser L.M., Gray J.W. (2020). How Machine Learning Will Transform Biomedicine. Cell.

[11] Semmler G., Bachmayer S., Wernly S., Wernly B., Niederseer D., Huber-Schönauer U., Stickel F., Aigner E., Datz C. (2020). Nut consumption and the prevalence and severity of non-alcoholic fatty liver disease. PLoS ONE.

[12] Brenner H., Hoffmeister M., Stegmaier C., Brenner G., Altenhofen L., Haug U. (2007). Risk of progression of advanced adenomas to colorectal cancer by age and sex: Estimates based on 840 149 screening colonoscopies. Gut.

[13] Chen T., Guestrin C. (2016). XGBoost: A Scalable Tree Boosting System. Proceedings of the 22nd ACM SIGKDD International Conference on Knowledge Discovery and Data Mining.

[14] Waldmann E., Heinze G., Ferlitsch A., Gessi I., Sallinger D., Jeschek P., Britto-Arias M., Salzl P., Fasching E., Jilma B. (2016). Risk factors cannot explain the higher prevalence rates of precancerous colorectal lesions in men. Br. J. Cancer.

[15] Wernly S., Wernly B., Semmler G., Bachmayer S., Niederseer D., Stickel F., Huber-Schönauer U., Aigner E., Datz C. (2021). A sex-specific propensity-adjusted analysis of colonic adenoma detection rates in a screening cohort. Sci. Rep..

[16] Doubeni C.A., Corley D.A., Quinn V.P., Jensen C.D., Zauber A.G., Goodman M., Johnson J.R., Mehta S.J., Becerra T.A., Zhao W.K. (2018). Effectiveness of screening colonoscopy in reducing the risk of death from right and left colon cancer: A large community-based study. Gut.

[17] Ran T., Cheng C.-Y., Misselwitz B., Brenner H., Ubels J., Schlander M. (2019). Cost-Effectiveness of Colorectal Cancer Screening Strategies—A Systematic Review. Clin. Gastroenterol. Hepatol..

[18] Nishihara R., Wu K., Lochhead P., Morikawa T., Liao X., Qian Z.R., Inamura K., Kim S.A., Kuchiba A., Yamauchi M. (2013). Long-Term Colorectal-Cancer Incidence and Mortality after Lower Endoscopy. N. Engl. J. Med..

[19] Lin J.S., Piper M.A., Perdue L.A., Rutter C.M., Webber E.M., O’Connor E., Smith N., Whitlock E.P. (2016). Screening for Colorectal Cancer: Updated Evidence Report and Systematic Review for the US Preventive Services Task Force. JAMA.

[20] Jones R.M., Devers K.J., Kuzel A.J., Woolf S.H. (2010). Patient-Reported Barriers to Colorectal Cancer Screening: A Mixed-Methods Analysis. Am. J. Prev. Med..

[21] Ferlitsch M., Reinhart K., Pramhas S., Wiener C., Gal O., Bannert C., Hassler M., Kozbial K., Dunkler D., Trauner M. (2011). Sex-Specific Prevalence of Adenomas, Advanced Adenomas, and Colorectal Cancer in Individuals Undergoing Screening Colonoscopy. JAMA.

[22] Ladabaum U., Mannalithara A., Meester R.G., Gupta S., Schoen R.E. (2019). Cost-Effectiveness and National Effects of Initiating Colorectal Cancer Screening for Average-Risk Persons at Age 45 Years Instead of 50 Years. Gastroenterology.

[23] Choi K.S., Lee H.-Y., Jun J.K., Shin A., Park E.-C. (2012). Adherence to follow-up after a positive fecal occult blood test in an organized colorectal cancer screening program in Korea, 2004-2008. J. Gastroenterol. Hepatol..

[24] Thomas M., Sakoda L.C., Hoffmeister M., Rosenthal E.A., Lee J.K., van Duijnhoven F.J., Platz E.A., Wu A.H., Dampier C.H., de la Chapelle A. (2020). Genome-wide Modeling of Polygenic Risk Score in Colorectal Cancer Risk. Am. J. Hum. Genet..

[25] Werner S., Krause F., Rolny V., Strobl M., Morgenstern D., Datz C., Chen H., Brenner H. (2016). Evaluation of a 5-Marker Blood Test for Colorectal Cancer Early Detection in a Colorectal Cancer Screening Setting. Clin. Cancer Res..

[26] Niehous K., Wan N., White B., Kannan A., Gafni E., Liu T.-Y., Haque I., Putcha G. (2018). Early Stage Colorectal Cancer Detection Using Artificial Intelligence and Whole-Genome Sequencing of Cell-Free DNA in a Retrospective Cohort of 1040 Patients. Am. J. Gastroenterol..

[27] Ternes D., Karta J., Tsenkova M., Wilmes P., Haan S., Letellier E. (2020). Microbiome in Colorectal Cancer: How to Get from Meta-omics to Mechanism?. Trends Microbiol..

[28] Aleksandrova K., Reichmann R., Kaaks R., Jenab M., Bueno-De-Mesquita H.B., Dahm C.C., Eriksen A.K., Tjønneland A., Artaud F., Boutron-Ruault M.-C. (2021). Development and validation of a lifestyle-based model for colorectal cancer risk prediction: The LiFeCRC score. BMC Med..

[29] Click B., Pinsky P.F., Hickey T., Doroudi M., Schoen R.E. (2018). Association of Colonoscopy Adenoma Findings With Long-term Colorectal Cancer Incidence. JAMA.

[30] Khera R., Haimovich J., Hurley N.C., McNamara R., Spertus J.A., Desai N., Rumsfeld J.S., Masoudi F.A., Huang C., Normand S.-L. (2021). Use of Machine Learning Models to Predict Death After Acute Myocardial Infarction. JAMA Cardiol..

