# Supplementary material for: Machine Learning Models Cannot Replace Screening Colonoscopy for the Prediction of Advanced Colorectal Adenoma
Source: J Pers Med. 2021 Sep 29;11(10):981. doi: 10.3390/jpm11100981 (PMC8538127; doi:10.3390/jpm11100981)
Supplement: Supplementary file 1 [file jpm-11-00981-s001.zip › jpm-1371930-supplementary.pdf]

SUPPLEMENTARY MATERIAL

Supplementary methods

Questionnaire on dietary habits

The food-frequency-questionnaire consisted of 69-items and conducted in line with the FFQ used in the SAPHIR (Salzburg Atherosclerosis Prevention Program in Subjects at High Individual Risk) study (1), as well as the EPIC study (2-4) counting total number of portions for each food type per week, or categorically reporting their frequency between <1 time/month and 4-5 times/day, depending on the food idem. Data on 8 food types/dietary patterns including alcohol consumption, coffee consumption, red meat consumption, and consumption of SSB, fruits, vegetables and fast-food were extracted as previously described (5). Specifically, alcohol consumption, coffee consumption and consumption of SSB were assessed quantitatively as drinks/week, while red meat consumption was assessed quantitatively as main dishes/week. Consumption of fruits, vegetables and fast-good were semi-quantitatively assessed based on six categories.

Definitions

Components of the metabolic syndrome were defined according to the IDF/AHA/NHLBI consensus definition (6): Visceral obesity was defined as waist circumference ≥88cm for women and ≥102cm for men. Hypertension was defined as a blood pressure ≥130/85mmHg on two consecutive days, or current use of antihypertensive medication. Dyslipidemia was defined as high-density lipoprotein cholesterol (HDL-cholesterol) <40mg/dl in males or <50mg/dl in females, triglycerides ≥150mg/dl or current use of antilipidemic drugs. The metabolic syndrome was defined as any three of the following: Visceral obesity, hypertension, fasting blood glucose ≥100mg/dl or antidiabetic therapy, HDL-cholesterol <40mg/dl in males or <50mg/dl in females or antilipidemic drugs, and triglycerides ≥150mg/dl. Diabetes was defined as either a blood glucose of ≥200mg/dl after 2 hours following oral glucose tolerance test or fasting blood glucose ≥126mg/dl, HbA1c ≥6.5%, or previously prescribed antidiabetic medication including insulin (7). Hepatic steatosis was assessed based on ultrasound.

Supplementary results

Supplementary Table S1. Detailed characteristics of the logistic regression (LR) and XGBoost-based models to predict advanced adenoma (AA).

	AUC	Sensitivity	Specificity	PPV	NPV
**LR**	0.66 (0.650-0.663)	15% (14.2-16.6%)	93% (92.7-93.3%)	15% (14.3-15.7%)	93% (93.1-93.3%)
**XGB**	0.66 (0.658-0.668)	14% (12.8-14.5%)	93% (92.8-93.3%)	14% (13.1-14.7%)	93% (92.9-93.1%)

The following linear variables were included: age (years), systolic blood pressure (mmHg), diastolic blood pressure (mmHg), weight (kg), height (cm), BMI (kg/m^2), ALT (U/L), AST (UL), AP (U/L), GGT (U/L), bilirubin (mg/dL), cholinesterase (U/L), uric acid (mg/dL), cholesterol(mg/dL), HDL-cholesterol (mg/dL), LDL-cholesterol (mg/dL), triglycerides (mg/dL), fasting blood glucose (mg/dL), thyroid-stimulating hormone (µU/ml), international normalized ratio, lipase (U/L), amylase (U/L), hemoglobin (g/dL), platelet count (G/L), C-reactive protein (mg/dL), blood sedation rate (seconds), LDH (U/L), iron (µg/dL), ferritin (nmol/L), transferrin saturation (%), transferrin (mg/dL), albumin (mg/dL), serum-protein-pheresis, homeostatic model assessment for insulin resistance (HOMA-IR), oral glucose tolerance test after 2 hours (mg/dL), alcohol (g/day), coffee (drinks/week), red meat (main dishes/week), SSB (drinks/week). The following categorical variables were included: Sex, smoking status, family history of CRC, acetylsalicylic acid intake, statin intake, ACE-inhibitor intake, hepatic steatosis, significant alcohol consumption, consumption of fast-food, consumption of fruits, consumption of vegetables.

Supplementary Table S2. Detailed characteristics of the logistic regression (LR) and XGBoost-based models to predict advanced adenoma (AA) when only selecting variables with suspected pathophysiological influence on AA risk.

	AUC	Sensitivity	Specificity	PPV	NPV
**LR**	0.66 [0.660-0.670]	16% (15.6-17.5%)	93% (92.3-93.1%)	16% (15.2-16.2%)	93% (93.1-93.3%)
**XGB**	0.65 [0.642-0.658]	17% (16.0-17.8%)	93% (92.2-93.1%)	15% (15.1-16.4%)	93% (93.1-93.3%)

The following linear variables were included: age (years), systolic blood pressure (mmHg), BMI (kg/m²), LDL-cholesterol (mg/dL), fasting blood glucose (mg/dL), C-reactive protein (mg/dL), alcohol (g/day). The following categorical variables were included: Sex, smoking status, family history of CRC.

Supplementary Table S3. Detailed characteristics of the logistic regression (LR) and XGBoost-based models to predict advanced adenoma (AA) when only selecting patients without family history.

	**AUC**	**Sensitivity**	**Specificity**	**PPV**	**NPV**
**LR**	0.63 (0.615-0.635)	13% (12.0-13.7%)	93% (93.6-94.0%)	14% (13.2-14.5%)	93% (93.1-93.3%)
**XGB**	0.64 (0.636-0.655)	14% (12.5-14.5%)	93% (93.0-93.8%)	15% (13.2-16.3%)	93% (93.1-93.3%)

The same variables were included in this model as for Supplementary Table 2.

Supplementary Table S4. Detailed characteristics of the logistic regression (LR) and XGBoost-based models to predict advanced adenoma (AA) when only selecting patients in specific age range between 45 and 80 at the time of examination.

	AUC	Sensitivity	Specificity	PPV	NPV
**LR**	0.66 (0.656-0.668)	14% (12.8-15.5%)	93% (92.6-93.2%)	14% (13.0-15.0%)	93% (92.8-93.1%)
**XGB**	0.65 (0.640-0.652)	14% (12.3-16.5%)	92% (92.1-92.9%)	13% (12.0-14.0%)	93% (92.7-93.1%)

The same variables were included in this model as for Supplementary Table 2.

Supplementary Table S5. Detailed characteristics of the logistic regression (LR) and XGBoost-based models to predict advanced adenoma (AA) when separating male and female cohorts.

Male:

	**AUC**	**Sensitivity**	**Specificity**	**PPV**	**NPV**
**LR**	0.58 (0.566-0.589)	14% (12.7-16.0%)	92% (91.5-92.3%)	16% (14.9-17.1%)	90% (90.5-90.9%)
**XGB**	0.60 (0.587-0.604)	14% (12.6-16.2%)	90% (89.6-90.6%)	14% (12.2-15.3%)	90% (90.3-90.7%)

Female:

	**AUC**	**Sensitivity**	**Specificity**	**PPV**	**NPV**
**LR**	0.59 (0.579-0.604)	1% (0.5-1.5%)	98% (98.5-99.0%)	0.5% (~0.0-0.5%)	95% (95.1-95.3%)
**XGB**	0.63 (0.603-0.658)	2% (1.1-2.5%)	99% (99.2-99.4%)	10% (6.4-13.5%)	95% (95.2-95.3%)

The same variables were included in this model as for Supplementary Table 2.

Supplementary Table S6. Detailed characteristics of the logistic regression (LR) and XGBoost-based models to predict advanced adenoma (AA) when excluding individuals with any adenoma others than AA.

	AUC	Sensitivity	Specificity	PPV	NPV
**LR**	0.70 (0.693-0.711)	28% (26.7-29.2%)	90% (90.0-90.9%)	24% (23.5-25.4%)	92% (91.8-92.1%)
**XGB**	0.70 (0.685-0.709)	28% (26.2-30.8%)	89% (89.0-89.5%)	22% (21.0-23.7%)	92% (91.6-92.1%)

The same variables were included in this model as for Supplementary Table S2.

References used in the supplementary materials

1. Kiechl S, Pechlaner R, Willeit P, et al. Higher spermidine intake is linked to lower mortality: a prospective population-based study. Am J Clin Nutr 2018;108:371-380.
2. Bohlscheid-Thomas S, Hoting I, Boeing H, et al. Reproducibility and relative validity of food group intake in a food frequency questionnaire developed for the German part of the EPIC project. European Prospective Investigation into Cancer and Nutrition. Int J Epidemiol 1997;26 Suppl 1:S59-70.
3. Bohlscheid-Thomas S, Hoting I, Boeing H, et al. Reproducibility and relative validity of energy and macronutrient intake of a food frequency questionnaire developed for the German part of the EPIC project. European Prospective Investigation into Cancer and Nutrition. Int J Epidemiol 1997;26 Suppl 1:S71-81.
4. Boeing H, Wahrendorf J, Becker N. EPIC-Germany--A source for studies into diet and risk of chronic diseases. European Investigation into Cancer and Nutrition. Ann Nutr Metab 1999;43:195-204.
5. Semmler G, Bachmayer S, Wernly S, et al. Nut consumption and the prevalence and severity of non-alcoholic fatty liver disease. PLoS One 2020;15:e0244514.
6. Alberti KG, Eckel RH, Grundy SM, et al. Harmonizing the metabolic syndrome: a joint interim statement of the International Diabetes Federation Task Force on Epidemiology and Prevention; National Heart, Lung, and Blood Institute; American Heart Association; World Heart Federation; International Atherosclerosis Society; and International Association for the Study of Obesity. Circulation 2009;120:1640-5.
7. Diagnosis and classification of diabetes mellitus. Diabetes Care 2014;37 Suppl 1:S81-90.
